# Arabidopsis INHIBITOR OF GROWTH 2 promotes flowering by regulating NuA4-dependent H4 acetylation levels at *FT* and *SOC1*

**DOI:** 10.1093/plphys/kiaf511

**Published:** 2025-10-16

**Authors:** Javier Barrero-Gil, Alfonso Mouriz, Raquel Piqueras, Yingnan Tian, Juan A López, Jesús Vázquez, Pedro Crevillén, José A Jarillo, Manuel Piñeiro

**Affiliations:** Centro de Biotecnología y Genómica de Plantas (CBGP), Universidad Politécnica de Madrid (UPM)—Instituto Nacional de Investigación y Tecnología Agraria y Alimentaria (INIA/CSIC), Pozuelo de Alarcón (Madrid), 28223, Spain; Centro de Biotecnología y Genómica de Plantas (CBGP), Universidad Politécnica de Madrid (UPM)—Instituto Nacional de Investigación y Tecnología Agraria y Alimentaria (INIA/CSIC), Pozuelo de Alarcón (Madrid), 28223, Spain; Centro de Biotecnología y Genómica de Plantas (CBGP), Universidad Politécnica de Madrid (UPM)—Instituto Nacional de Investigación y Tecnología Agraria y Alimentaria (INIA/CSIC), Pozuelo de Alarcón (Madrid), 28223, Spain; Centro de Biotecnología y Genómica de Plantas (CBGP), Universidad Politécnica de Madrid (UPM)—Instituto Nacional de Investigación y Tecnología Agraria y Alimentaria (INIA/CSIC), Pozuelo de Alarcón (Madrid), 28223, Spain; Centro Nacional de Investigaciones Cardiovasculares (CNIC), Madrid 28029, Spain; CIBER de Enfermedades Cardiovasculares (CIBERCV), Madrid 28029, Spain; Centro Nacional de Investigaciones Cardiovasculares (CNIC), Madrid 28029, Spain; CIBER de Enfermedades Cardiovasculares (CIBERCV), Madrid 28029, Spain; Centro de Biotecnología y Genómica de Plantas (CBGP), Universidad Politécnica de Madrid (UPM)—Instituto Nacional de Investigación y Tecnología Agraria y Alimentaria (INIA/CSIC), Pozuelo de Alarcón (Madrid), 28223, Spain; Centro de Biotecnología y Genómica de Plantas (CBGP), Universidad Politécnica de Madrid (UPM)—Instituto Nacional de Investigación y Tecnología Agraria y Alimentaria (INIA/CSIC), Pozuelo de Alarcón (Madrid), 28223, Spain; Centro de Biotecnología y Genómica de Plantas (CBGP), Universidad Politécnica de Madrid (UPM)—Instituto Nacional de Investigación y Tecnología Agraria y Alimentaria (INIA/CSIC), Pozuelo de Alarcón (Madrid), 28223, Spain

## Abstract

INHIBITOR OF GROWTH (ING) proteins are chromatin readers that bind trimethylated histone H3 lysine (K) 4 (H3K4me3) marks and associate with either histone acetyltransferase or deacetylase complexes to activate or repress gene transcription, respectively. In plants, there are two types of ING proteins, namely ING1 and ING2. Here, we report that *Arabidopsis thaliana* ING2 associates with multiple subunits of the histone H4 acetyltransferase complex NuA4, controls genome-wide levels of histone H4 acetylation (H4ac), and regulates different developmental processes, including the initiation of flowering. Our data indicate that ING2 biological functions are largely independent of ING1 activity. We find that ING2 is recruited to the chromatin of key floral integrators, such as *FT* and *SOC1*, and is required for their timely activation by modulating histone H4ac levels at these loci. Our observations reveal a genetic interaction between *ING2* and genes encoding relevant proteins for H3K4me3 or H2A.Z deposition, suggesting that ING2 might represent a hub for potential crosstalk between histone H4ac and these histone modifications/variants.

## Teaser

INHIBITOR OF GROWTH 2 promotes flowering by regulating NuA4-dependent histone H4 acetylation levels at floral integrator genes in Arabidopsis.

## Introduction

Histones are core components of chromatin and play a fundamental role in DNA functional biology. These proteins determine chromatin compaction and accessibility (Luger et al. 1997), which profoundly affects DNA transcription and replication. Histone post-translational modifications (PTMs) have been shown to be instrumental for such processes (Strahl and Allis 2000; Jenuwein and Allis 2001). Histone PTMs are recognized by specific proteins, known as histone readers (Kutateladze 2011), to trigger the recruitment of protein complexes at specific genomic loci conveying the information contained in histone PTMs (Bannister and Kouzarides 2011).

The plant homeodomain (PHD) finger is a zinc-binding domain of 50 to 80 amino acids (Mouriz et al. 2015) that enables reader binding to di- and tri-methylated histone H3 lysine 4 residues (H3K4me2/me3), landmarks of transcriptionally active genes (Quan et al. 2023). PHD finger proteins have emerged as pivotal epigenetic readers and modulators in biology, orchestrating a broad spectrum of cellular processes in eukaryotes (Sanchez and Zhou 2011). Among the best known PHD-containing readers are the highly conserved INHIBITOR OF GROWTH (ING) proteins (He et al. 2005), which are characterized not only by their capacity to bind methylated histone H3, but also phosphoinositol monophosphates, and to regulate gene expression through the recruitment of histone acetyltransferases (HAT) and histone deacetylases (HDACs) complexes to activate or repress gene expression respectively (Soliman and Riabowol 2007). The ING family in mammals (ING1-5) comprises five members that play crucial roles in DNA damage repair, cell apoptosis, and tumor suppression (Taheri et al. 2022). However, phylogenetic analysis indicates that plant ING proteins group in two clades, each one representing ING1-like and ING2-like proteins (Jaudal et al. 2022). The genomes of Arabidopsis (Lee et al. 2009) and Medicago (Jaudal et al. 2022) contain a representative of each group and both display the capacity to bind the active transcription epigenetic mark H3K4me3 in vitro (Lee et al. 2009; Zhao et al. 2018 ). Plant ING proteins may associate with several chromatin-modifying complexes. In Arabidopsis and rice, ING1 was recently shown to recruit a plant-specific GENERAL CONTROL NON-DEPRESSIBLE 5 (GCN5)-containing complex called Plant-ADA2A-GCN5 Acetyltransferase (PAGA) that catalyses the acetylation of histone H3 (Wu et al. 2023). GCN5 HAT functions in both Spt-Ada-Gcn5 Acetyltransferase (SAGA) and PAGA complexes to activate gene expression by stimulating H3K9/K14Ac in promoter regions (Gan et al. 2021). Arabidopsis *ing1* mutants display a moderate reduction in height and show higher numbers of primary branches in comparison to Col plants, without altering the flowering time phenotype (Wu et al. 2023).

On the other hand, proteomic studies and protein–protein binding assays in Arabidopsis have shown that ING2 proteins interact with different chromatin remodeling factors including HDACs like HDC1 (Perrella et al. 2016) and NuA4-C (for Nucleosome Acetyltransferase of H4) subunits (Tan et al. 2018; Barrero-Gil et al. 2022; Bieluszewski et al. 2022). In yeast, NuA4 bears three main modules with a central core and two lobes. The central core contains the Transcription associated protein (Tra1), the EAF1 protein and the Piccolo module (with ESA1, YNG2, ENHANCER OF POLYCOMB-LIKE 1 (EPL1) and ESA1-associated factor 6 (EAF6) subunits), while lobe 1 is formed by the Trimer Independent of NuA4 involved in Transcription Interactions with Nucleosomes (TINTIN) sub-module, and lobe 2 comprises the four subunits shared with SWR1-C (SWC4, YAF9, ARP4, and ACT1) involved in the exchange of histone H2A by the H2A.Z variant (Setiaputra et al. 2018). However, whether plant ING2 proteins are core subunits of these complexes remains unclear, and no proteomic studies using ING2 as a bait have been reported so far. ING2 promotes flowering in Medicago (Jaudal et al. 2022), though the exact mechanism remains to be elucidated. Similarly, NuA4-C is known to promote flowering in Arabidopsis through H2A.Z (Crevillen et al. 2019) and H4 acetylation (Xiao et al. 2013; Barrero-Gil et al. 2021). Therefore, it is tempting to speculate that ING2 could regulate flowering through the modulation of NuA4-C-histone acetylation activity. In addition, whether ING2 recruits additional protein complexes remains an open question.

In this study, using Arabidopsis as genetic model, we take unbiased proteomics approaches to unveil ING2-bound proteins, we analyze the relationship between the two Arabidopsis ING proteins, characterize the physiological role of ING2 and study the molecular mechanism that enables ING2 to regulate flowering. Our results demonstrate that ING2 is part of NuA4-C, and acts modulating genome-wide histone H4ac levels and controlling different developmental responses including flowering time. Our results argue for distinct epigenetic mechanisms mediating the function of the two plant types of ING readers in regulating gene expression. In particular, Arabidopsis ING2 controls flowering time through the regulation of H4ac levels at chromatin loci encoding key flowering time regulators.

## Results

### ING2 is co-purified along with most subunits of NuA4-C

We followed AP-MS approaches to identify the proteins that can co-purify with the ING2 protein in aerial tissues from 12 day-old seedlings grown under long days (LD) and 22 °C. We identified among others, peptides corresponding to 8 out of the 12 subunits that are thought to constitute the plant NuA4-C (Espinosa-Cores et al. 2020), including proteins from the Piccolo (HAM1, EPL1A, EPL1B, and EAF6), YEATS (YAF9A, SWC4, and ARP4) and core modules (EAF1A, EAF1B, TRA1A, and TRA1B) (Table 1  Supplementary Fig. S1, A and Supplementary Table S1), while no peptide corresponding to any subunit from the TINTIN module (EAF3 and EAF7) co-purified with ING2 (Doyon and Cote 2004). These results together with our previous data (Barrero-Gil et al. 2022) and data published by other groups (Bieluszewski et al. 2015; Tan et al. 2018; Bieluszewski et al. 2022; Zhou et al. 2022; Zheng et al. 2023) firmly establish ING2 as a bona-fide member of the NuA4-C (Supplementary Fig. S1A). Remarkably, we failed to detect peptides from any HDAC complex subunits (Supplementary Table S1). We used the protein–protein interaction database STRING (https://string-db.org/) to further analyze ING2 co-purifying proteins, finding protein networks related to pre-mRNA processing factor 19 (PRP19) (Chanarat and Strasser 2013), Mediator (Buendia-Monreal and Gillmor 2016) and the NuA4 complexes (Supplementary Table S1, Supplementary Fig. S1B). A functional enrichment analysis of this network revealed terms related to H4 acetylation and mRNA splicing.

**Table 1.** ING2 interacts with NuA4 complex subunits

Uniprot accession	Mol. weight	AGI	Name	Description	Control		myc-ING2	
					#1	#2	#1	#2
ARP4_ARATH	49 kDa	At1g18450	ARP4	Actin-related protein 4	0	0	18	39
Q8GYQ9_ARATH	52 kDa	At1g79020	EPL1B	Enhancer of polycomb-like protein 1	0	0	15	30
A0A178VRI9_ARATH	50 kDa	At2g47210	SWC4	myb-like transcription factor family protein	0	0	13	32
EAF1B_ARATH	206 kDa	At3g24870	EAF1B	Chromatin modification-related protein EAF1B	0	0	12	29
EAF1A_ARATH	212 kDa	At3g24880	EAF1A	Chromatin modification-related protein EAF1A	0	0	12	27
F4HWW1_ARATH	37 kDa	At1g54390	ING2	Inhibitor of growth 2	0	0	18	14
A0A178UAU6_ARATH	51 kDa	At5g64610	HAM1	Histone acetyltransferase	0	0	1	22
TAF14B_ARATH	30 kDa	At5g45600	YAF9A	Transcription initiation factor TFIID subunit 14b	0	0	6	16
Q93VF4_ARATH	18 kDa	At4g14385	EAF6	Histone acetyltransferase subunit NuA4-domain	0	0	8	13
A0A178UUC0_ARATH	434 kDa	At4g36080	TRA1B	Phosphatidylinositol kinase	0	0	1	19
Q9FX82_ARATH	51 kDa	At1g16690	EPL1A	Enhancer of polycomb-like protein 1	0	0	0	6
A0A178VNS2_ARATH	436 kDa	At2g17930	TRA1A	Phosphatidylinositol 3- and 4-kinase family protein	1	0	1	6

List of NuA4-C subunits identified among proteins co-purified with the ING2 protein in this study.

### ING2 regulates flowering time and additional developmental processes

**Figure 1.**
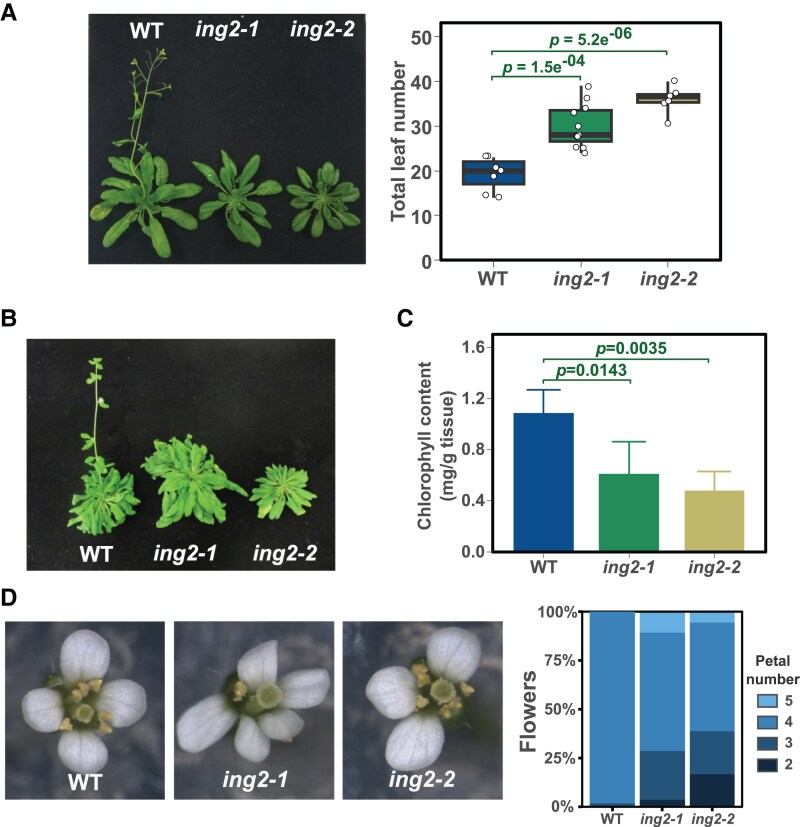
Arabidopsis *ING2* is involved in the promotion of flowering and additional developmental processes. **A-B)** Flowering time in *ing2* mutant plants under long-day **(A)** or short-day **(B)** photoperiod. Quantification under SD is not feasible since most of the plants do not flower in these conditions. **C-D)** Analysis of chlorophyll content **(C)** and flower development **(D)** in *ing2* mutant plants. Box plots **(A)** show the interquartile range of the data and the median is indicated by a line. Whiskers represent the minimum and maximum value. Individual data points are displayed by white dots. Bar plots **(C)** show average and standard deviation from four biological replicates. Statistical significance is indicated providing *P*-value in a Dunnet's test **(A** and **C)**.

To unveil the functions carried out by the ING2 protein, we obtained from the Arabidopsis NASC germplasm collection two T-DNA mutant lines, GABI_166D07 (*ing2-1*) and GABI_909H04 (*ing2-2*), that were confirmed to be KO mutants for *ING2* (Supplementary Fig. S2, A and B). Loss-of-function alleles of *ING2* resulted in a delayed flowering time under LD (Fig. 1A), in line with observations made in Medicago (Jaudal et al. 2022). Intriguingly, *ing2* mutant plants grown under short days (SD) did not flower, entering senescence without bolting or setting seeds (Fig. 1B). Additionally, over-expression of *ING2* complemented the late flowering phenotype observed in the *ing2* knock-out (KO) mutants (Supplementary Fig. S3), confirming that ING2 is a positive regulator of the floral transition. Further analysis showed that the loss of *ING2* function also affects chlorophyll accumulation (Fig. 1C) and flower architecture, altering the number of petals (Fig. 1D).

### ING2 biological functions are largely independent of ING1 activity

**Figure 2.**
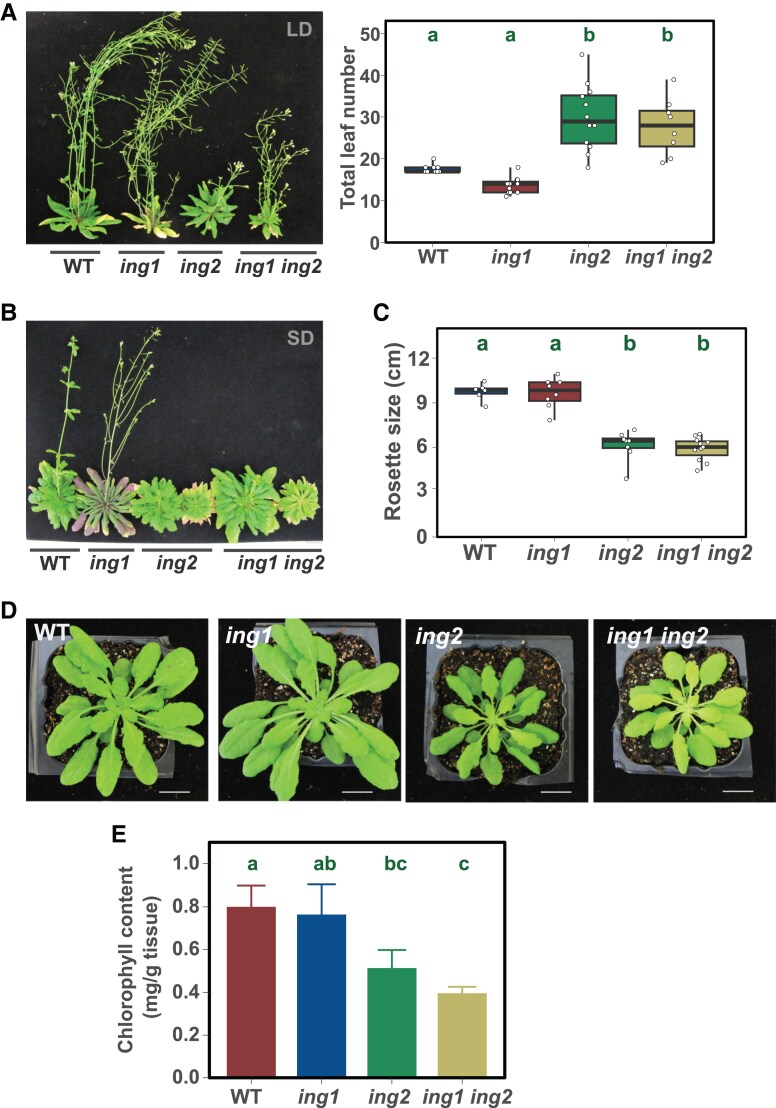
Developmental alterations in *ing2* mutant plants are independent of *ING1* function. **A**-**B)** Genetic relationship of *ING1* and *ING2* genes in the determination of flowering time under long-day (A) or short-day (B) photoperiod. **C)** Quantification of rosette diameter in plants grown under SD. **D)** Representative plants of the indicated genotypes grown under non-inductive photoperiodic conditions. **E)** Determination of chlorophyll content in 10 day-old seedlings grown under LD. Box plots **A, C)** show the interquartile range of the data and the median is indicated by a line. Whiskers represent the minimum and maximum value. Individual data points are displayed by white dots. Bar plots (E) show average and standard deviation from three biological replicates. Statistical significance in one-way ANOVA tests at *P* < 0.05 followed by Tukey's HSD test is indicated by different letters.

The Arabidopsis genome encodes two closely related ING proteins (Lee et al. 2009). To study the functional relationship between these two genes, we isolated *ing1* loss-of-function mutants (Wu et al. 2023) to generate *ing1 ing2* double mutant plants and compared flowering time with *ing1* and *ing2* single mutant plants. The results reveal opposite effects of the loss of *ING1* and *ING2* function on the determination of flowering time as *ing1* mutant plants tend to flower slightly earlier than wild-type (WT) under LD, while *ing2* mutants flower much later (Fig. 2A). Double *ing1 ing2* mutant plants also display a late flowering phenotype similar to the *ing2* single mutant plants. Under SD, the *ing1* mutant plants did not display obvious alterations in flowering time while both *ing2* and *ing1 ing2* double mutant plants failed to flower (Fig. 2B). In addition, we observed that *ing2* mutants showed smaller rosettes in these conditions regardless of the presence of an active ING1 protein (Fig. 2, C and D). Moreover, *ing1* mutants did not show altered levels of chlorophyll in clear contrast to *ing2* and *ing1 ing2* mutants that showed a similar reduction in chlorophyll content under LD photoperiod (Fig. 2E). Finally, we observed that the *ing1 ing2* double mutant produced shorter siliques under LD photoperiod (Supplementary Fig. S4). We measured silique length in single and double *ing* mutants, unveiling a synergistic effect of these mutations on the length of siliques (Supplementary Fig. S4). These results indicate that ING2 function in the regulation of flowering time, rosette size or the chlorophyll content, is not significantly affected by the loss of *ING1* function, while a more complex relationship between *ING* genes can be observed in the regulation of other developmental traits like fruit development, where loss-of-function of both genes results in an enhancement of the phenotypic alterations observed.

**Figure 3.**
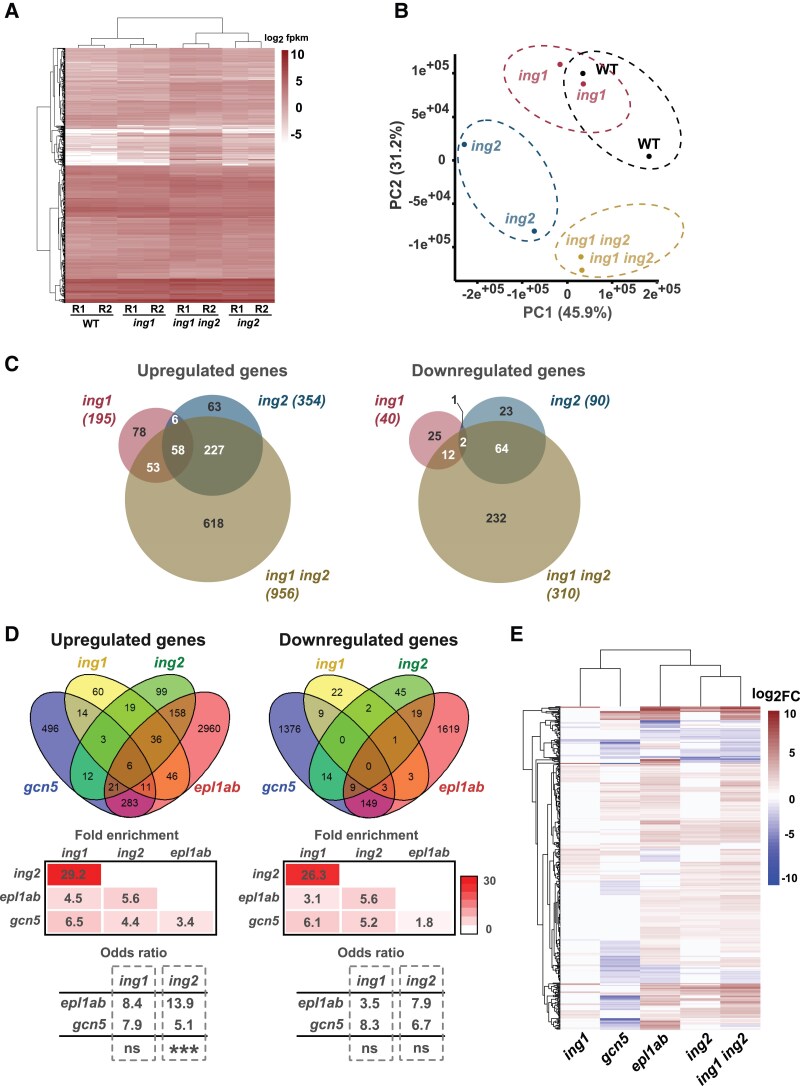
ING1 and ING2 mostly regulate different gene sets. **A)** Heatmap showing expression level of DEGs in *ing* mutants. **B)** Principal component analysis of expressed genes in the indicated genotypes. **C)** Overlap among DEGs in *ing* mutants. **D)** Overlap between ING-regulated genes and genes regulated by NuA4-C (*epl1ab*) (Barrero-Gil et al. 2022), and PAGA-C (*gcn5*) (Wu et al. 2023). Venn diagrams illustrate overlapping genes while heatmap shows the fold enrichment for each pairwise comparison denoted by color scale and the number on each panel. The odds ratio for each overlap is indicated below along with the result of z-test comparisons between encircled overlaps (ns, not significant, *** *P* < 0.001). **E)** Hierarchical clustering of gene expression profiles obtained for the mutants analyzed. The panel shows heatmaps comparing fold changes in gene expression in the indicated mutants. The analysis was performed with DEGs common to at least three analyzed mutants.

To further study the relationship between ING1 and ING2 proteins we compared global gene expression profiles in WT, *ing* single and double mutant seedlings. We detected 195, 354 and 956 upregulated genes, and 40, 90, and 310 downregulated genes in the *ing1*, *ing2*, and *ing1 ing2* double mutant, respectively (Fig. 3, Supplementary Table S2). Interestingly, the hierarchical clustering analysis grouped *ing2* and *ing1 ing2* together (Fig. 3A). Furthermore, in a principal component analysis (PCA) the transcriptomic profile of *ing1* mutant plants is not clearly separated from that of WT plants in a graphical representation using the first two principal components (Fig. 3B). In contrast, the transcriptomic profiles of *ing2* single and double mutant plants are clearly separated from that of WT and *ing1* plants (Fig. 3B). Venn diagrams showed a significant but limited overlap of genes regulated by ING1 and ING2, particularly among downregulated genes (Fig. 3C). A comparison of differentially expressed genes (DEGs) in *ing* mutants with EPL1 (Barrero-Gil et al. 2022) and GCN5-regulated genes (Wu et al. 2023), representing NuA4-C and PAGA-dependent genes respectively, reveals that misregulated genes in *ing2* mutants are more enriched in NuA4-C-dependent genes than in PAGA-dependent genes, while the reverse is true for *ing1* mutants (Fig. 3D). We assessed the statistical significance of these associations calculating the odds ratio (OR) for each overlap, finding higher ORs between *ing2* and *epl1a epl1b* (*epl1ab*) than between *ing2* and *gcn5* in all cases. In contrast, *ing1*-*gcn5* OR was higher than *ing1*-*epl1ab* OR for downregulated genes. An analysis of the difference of log (OR) showed a more significant association between *ing2* and *epl1ab* compared to *gcn5* in a z-test for upregulated genes (Fig. 3D). A hierarchical clustering of gene expression profiles revealed that while *ing1* is grouped with *gcn5*, *ing2* is grouped with *epl1ab* (Supplementary Fig. S4E). A gene ontology (GO) enrichment analysis reveals different specific and shared biological terms among DEGs in *ing* mutants (Supplementary Fig. S5, Supplementary Table S2). Overall, genetic and transcriptomic analysis suggest that Arabidopsis ING proteins co-regulate a very limited set of genes, consistent with both proteins being part of different chromatin remodeling complexes.

### ING2-mediated regulation of the floral transition requires di- and tri-methylated H3K4 marks

**Figure 4.**
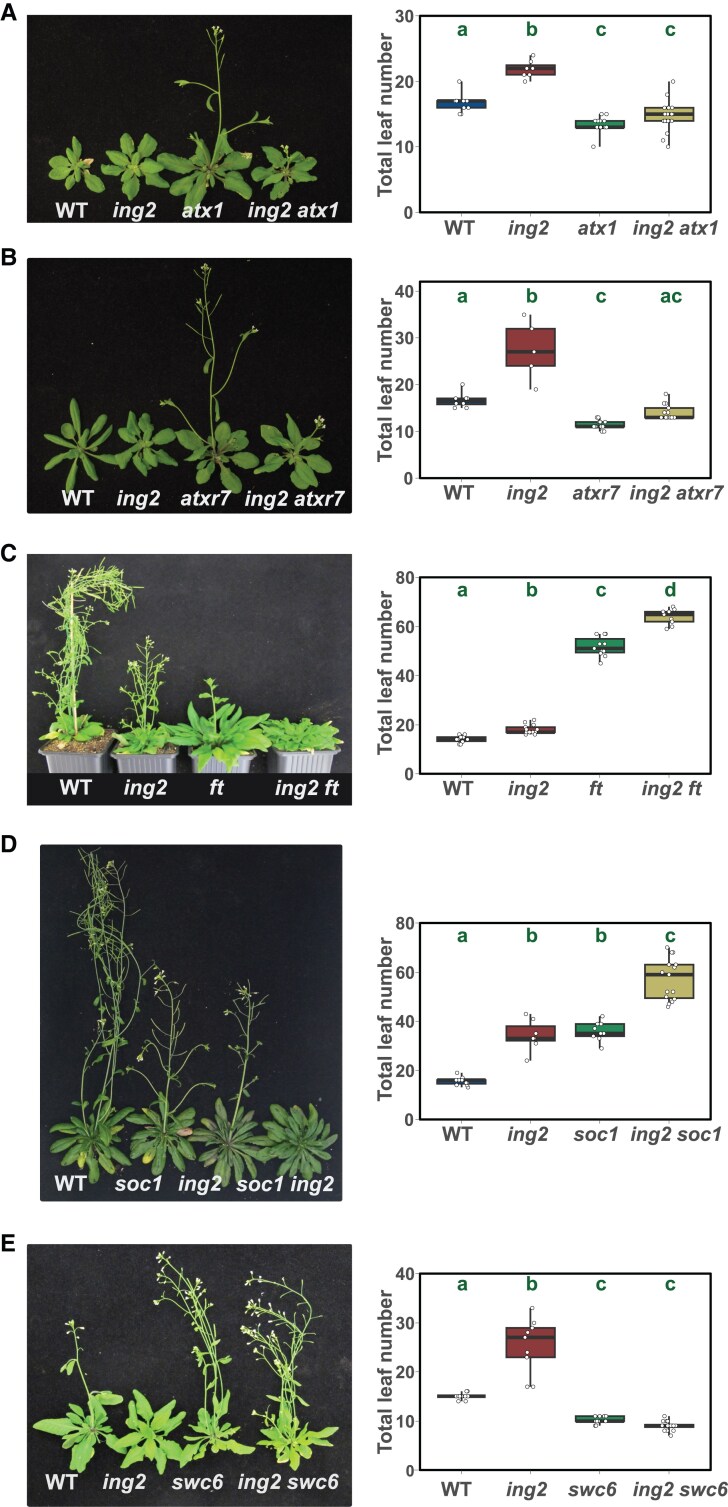
Genetic interactions provide insights into ING2 function in the regulation of flowering time. Flowering time was determined in single and double mutants deficient in the activities of *ING2* and *ATX1*  **(A)**, *ATXR7*  **(B)**, *FT*  **(C)**, *SOC1*  **(D)** or *SWC6*  **(E)** genes. Left panels show representative plants of the indicated genotypes. Right panels show flowering time scored as the number of total leaves. Plants were grown under LD photoperiodic conditions. Box plots **(A**-**E)** show the interquartile range of the data and the median is indicated by a line. Whiskers represent the minimum and maximum value. Individual data points are displayed by white dots. Statistical significance in one-way ANOVA tests at *P* < 0.05 followed by Tukey's HSD test is indicated by different letters.

Previous work has shown that Arabidopsis ING2 can bind the H3K4me2/me3 activating marks (Lee et al. 2009 ). Trithorax protein complexes are responsible for methylation of histone H3K4 residues in eukaryotes (Avramova 2009). The Arabidopsis genome includes ten Trithorax proteins (Tamada et al. 2009), and ARABIDOPSIS TRITHORAX1 (ATX1)/SET DOMAIN GROUP 27 (SDG27) (Pien et al. 2008; Jing et al. 2019) and ARABIDOPSIS TRITHORAX-RELATED7 (ATXR7)/SET DOMAIN GROUP 25 (SDG25) (Tamada et al. 2009) have been shown to regulate flowering time. We decided to explore genetic relationships of *ING2* with the genes encoding these two proteins by crossing the corresponding single loss-of-function mutant plants and analyzing the floral transition in the double mutant. The results show that the loss of function of either *ATX1* or *ATXR7* reverted the late flowering phenotype displayed by *ing2* mutant plants (Figs. 4, A and B), revealing a genetic interaction between these genes, which might suggest that *ING2* function depends on histone H3K4me2/me3 levels. However, further experiments will be needed to identify the genetic determinants underlying such interaction to firmly establish this association.

### The role of ING2 in flowering time control does not rely on the function of a single floral integrator and requires SWR1-C activity

Our data show that the ING2 protein interacts with a number of NuA4-C subunits and that it is necessary to promote flowering (Table 1 and Fig. 1). In previous works, NuA4-C was shown to regulate the initiation of flowering through the acetylation of histone H4 at *FLOWERING LOCUS T (FT)* and *SUPPRESSOR OF OVEREXPRESSION OF CONSTANS 1 (SOC1)* chromatin (Xu et al. 2014; Barrero-Gil et al. 2021) and H2A.Z at *FLOWERING LOCUS C (FLC)* chromatin (Crevillen et al. 2019). To address the mechanism by which ING2 regulates flowering, we crossed the *ing2* mutant with *ft* (Yoo et al. 2005) and *soc1* (Lee et al. 2000) mutants, affected in the central floral integrators *FT* and *SOC1*, respectively, and quantified their flowering time under a LD photoperiod regime (Figs. 4, C and D). The results show an additive effect of *ing2* and *ft* and *soc1* mutations, indicating that either ING2 role in flowering does not depend on a single floral integrator or such role is completely independent on *FT* and *SOC1* function.

Recent evidence suggests that NuA4-C may regulate the levels of the histone variant H2A.Z at certain genomic locations (Bieluszewski et al. 2022). To investigate a possible relationship between ING2 function and the deposition of the histone variant H2A.Z, we crossed the *ing2* mutant with the *swr1 complex 6* (*swc6*) mutant that is defective on the Swi2/Snf2-related chromatin remodeling complex SWR1 (SWR1-C) function and hence, on H2A.Z deposition on the chromatin (Lazaro et al. 2008). The *swc6* mutation causes plants to flower earlier than WT plants. The results show a clear epistatic relationship between *SWC6* and *ING2*, indicating that *ING2* function is dependent on SWR1-C activity (Fig. 4E). Altogether, our genetic analyses indicate that ING2 proteins integrate information relayed by histone H3K4me3 epigenetic marks, controlling flowering time possibly through several floral integrators. Such control is largely dependent on the activity of SWR1-C.

### ING2 promotes genome-wide histone H4 acetylation but not H2A.Z chromatin deposition

**Figure 5.**
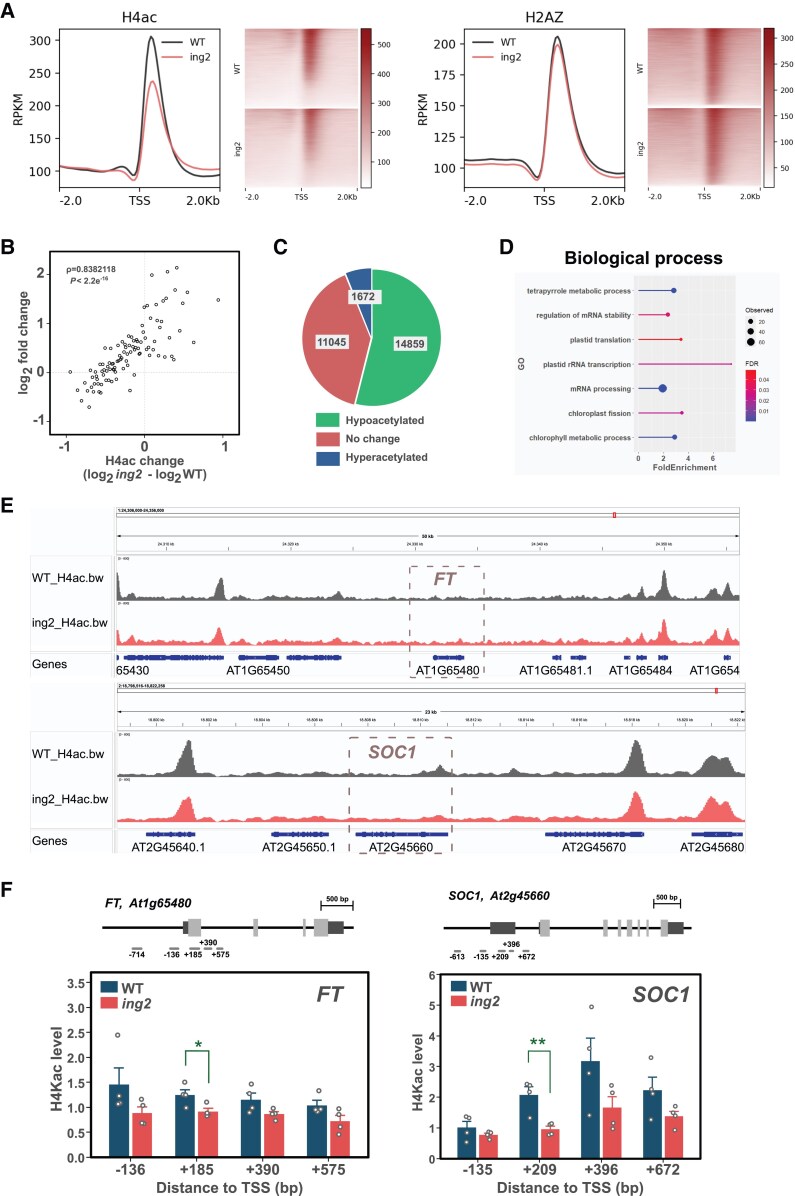
ING2 promotes global histone H4 acetylation but does not influence H2A.Z deposition. **A)** Metaplot and read density maps showing histone H4ac and H2A.Z signal level and distribution pattern in WT and *ing2* mutant plants. **B)** Spearman rank correlation analysis between changes in histone H4ac levels and gene expression observed in *ing2* mutant plants. Spearman coefficient and *P*-value are indicated in the inset within the scatter plot. **C)** Number of hypoacetylated and hyperacetylated peaks in *ing2* chromatin. **D)** Enrichment analysis of gene ontology terms associated with the hypoacetylated peaks identified in (C). **E)** Histone H4ac profiles of wild type (grey) and *ing2* (red) in genomic regions centered on *FT* and *SOC1* genes. Average signal from two replicates is shown. **F)** Histone H4ac levels at *FT* and *SOC1* genomic loci as determined by ChIP-qPCR experiments. Tested amplicons are indicated over each graph and below each gene scheme where coding regions of exons are depicted in light gray, and untranscribed regions (UTR) are shown in dark gray. Bars indicate the average of four biological replicates while error bars indicate SEM. Individual data points are displayed by white dots. Significant differences in two-sided t-tests are indicated with asterisks (* *P* < 0.05, ** *P* < 0.01).

Since the ING2 protein interacts with many NuA4-C subunits (Table 1), we anticipated that ING2 might contribute to the activity of this complex. To tackle this issue, we sequenced libraries prepared from chromatin immunoprecipitated (ChIP-seq) with antibodies raised against poly-acetylated histone H4 or unmodified histone H4 as a control. We also performed a ChIP-seq experiment to analyze chromatin H2A.Z levels, since previous observations proposed that NuA4-C promotes the deposition of this histone variant (Bieluszewski et al. 2022) and our genetic evidence showed an epistatic relationship between *ING2* and genes encoding proteins involved in histone H2A.Z deposition (Fig. 4). The results show a global decrease in histone H4 acetylation levels in *ing2* chromatin compared to WT but no significant changes in chromatin H2A.Z levels (Fig. 5A). Intriguingly, we also observed a reduction of histone H4 occupancy upstream and downstream of the transcriptional start site (TSS) (Supplementary Fig. S6A). To check if this circumstance could challenge our interpretation of the data, we normalized the histone H4ac and H2A.Z signals by histone H4 occupancy level reaching the same conclusions, i.e. ING2 promotes histone H4 acetylation but does not influence H2A.Z occupancy (Supplementary Fig. S6B). Next, we asked whether the altered levels of histone H4ac in the *ing2* mutant are associated with changes in gene expression. To do that, we averaged H4ac signal over a region spanning 500 base pairs downstream of the TSS for each gene and calculated the change in histone H4ac as the log_2_ difference between the *ing2* mutant and WT. We sorted genes according to histone H4ac change, divided them in percentiles and for each percentile, we averaged the histone H4ac change and gene expression level, which was derived from our transcriptomic analysis of *ing* mutants. Spearman rank correlation analysis revealed a significant association between histone H4ac and gene expression change in *ing2* mutant compared to WT (Fig. 5B). Next, we performed a differential peak enrichment analysis on our ChIP-seq data. The results show that the overwhelming majority of differentially acetylated genes correspond to genes with lower histone H4ac in the *ing2* mutant (Fig. 5C). Interestingly, we observed that ING2-mediated histone H4 acetylation is higher in H3K4me3-enriched genes, though this correlation appears to be absent for ING2-dependent H2A.Z deposition (Supplementary Fig. S7).

A GO analysis on the genes hypoacetylated in the *ing2* mutant revealed an enrichment of terms related to photosynthetic functions including plastid translation, chlorophyll biosynthesis and chloroplast fission (Fig. 5D). Interestingly, among the genes identified with lower H4ac levels we found diverse flowering integrators such as *SOC1* (Samach et al. 2000), *GIGANTEA* (*GI*) (Fowler et al. 1999) and *CONSTANS* (*CO*) (Putterill et al. 1995) but could not identify *FT* (Kardailsky et al. 1999), probably because of the low histone H4ac signal detected within this genomic region (Fig. 5E, Supplementary Table S3). We sought to confirm the results for *FT* and *SOC1* genes by performing ChIP-qPCR analysis. Remarkably, besides obtaining confirmatory data on the low histone H4ac status of *SOC1* chromatin, we also found a modest but significant reduction of *FT* locus histone H4ac level in the *ing2* mutant (Fig. 5F). In contrast, a similar approach did not reveal significant changes in histone variant H2A.Z levels at these loci in *ing2* plants (Supplementary Fig. S8).

Despite the absence of global changes in H2A.Z deposition in the *ing2* mutant, we performed a differential peak analysis to explore changes in H2A.Z occupancy that might account for the observed genetic interaction between *ING2* and *SWC6*. Intriguingly, we found 398 peaks with decreased H2A.Z occupancy, and 3021 peaks showing higher H2A.Z occupancy (Supplementary Fig. S9A, Supplementary Table S4). However, this differential H2AZ occupancy is only associated with differential gene expression in the *ing2* mutant in the case of repressed genes (Supplementary Fig. S9B). We investigated the connection between ING2-mediated NuA4 activity and H2A.Z occupancy. Though we did not find significant differences in H2A.Z enrichment in histone H4 hypo- or hyperacetylated genes, we found significant differences in NuA4-bound genes but the size effect was small (r = 0.03) (Supplementary Fig. S9C). In addition, we detected a higher histone H4ac level in genes with increased H2A.Z occupancy, also with a modest size effect (r = 0.08) (Supplementary Fig. S9D). In summary, our data support a broad effect of ING2 on promoting histone H4 acetylation with limited impact on H2A.Z occupancy. Intriguingly, the observed moderate effects of *ING2* function on H2A.Z enrichment are weakly associated with histone H4ac.

### ING2 regulates flowering time activating *FT* and *SOC1* transcription

**Figure 6.**
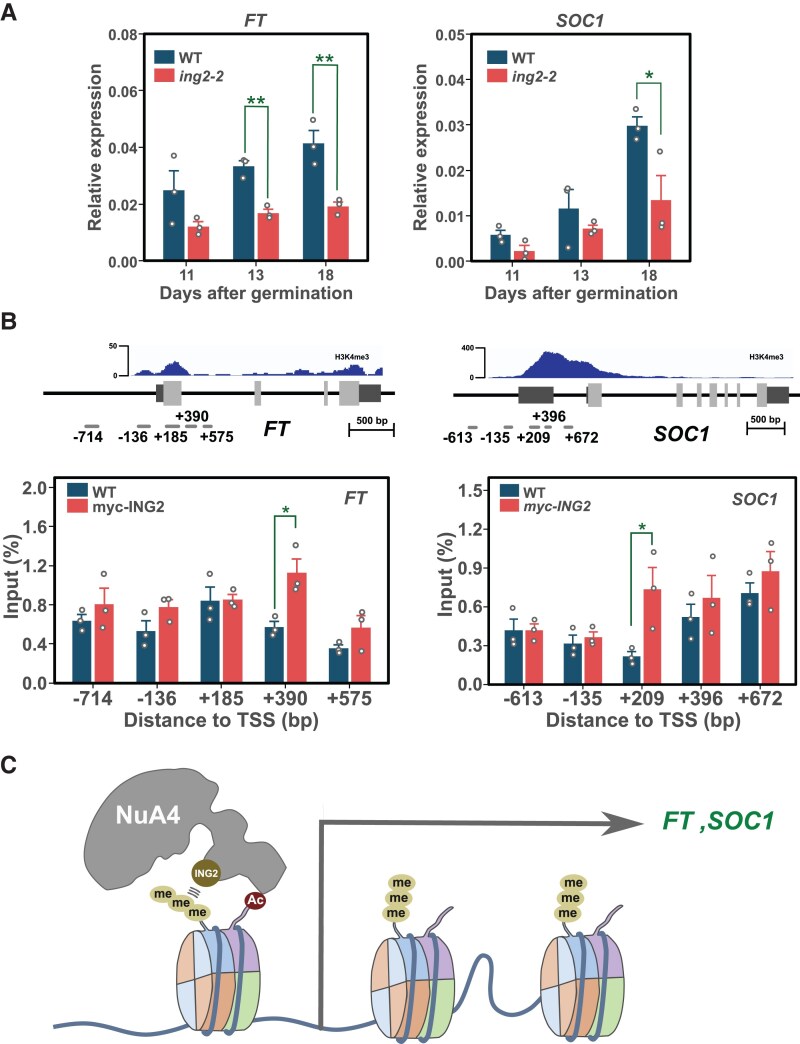
ING2 protein binds *FT* and *SOC1* chromatin to regulate histone H4ac level promoting the activation of the floral integrator genes. **A)** Developmental expression analysis of floral integrators in WT and *ing2* mutant. **B)** ChIP-qPCR experiments in WT and transgenic *35S-myc-ING2* plants. The data show the immunoprecipitated fraction recovered from *FT* or *SOC1* chromatin after immunoprecipitation with the anti-myc antibody. Coverage data for H3K4me3 in WT is indicated as a reference above gene structure. **C)** Hypothetical working model on ING2 function. As part of NuA4-C, ING2 subunit likely enables H3K4me3 reading and participates in the modulation of histone H4ac levels in floral integrator target genes *FT* and *SOC1*. A similar molecular mechanism might mediate the involvement of ING2 in the regulation of additional developmental processes that are altered in the *ing2* mutant by modulating acetylation levels in the chromatin of other target genes. Bars indicate the average of three biological replicates and error bars denote SEM. Individual data points are displayed by white dots. Asterisks denote significance differences in two-sided t-tests (* *P* < 0.05, ** *P* < 0.01).

To clarify if the low histone H4ac status on *FT* and *SOC1* chromatin in *ing2* mutants is correlated with a lower expression of these genes, we performed a time-course gene expression analysis of these floral integrator genes in the *ing2* mutant (Fig. 6A). The results obtained show a clear decrease in the activation of both flowering activators. To confirm that these genes might be direct targets of *ING2* regulation, we performed ChIP experiments with a transgenic line that overexpresses a myc-tagged construct of ING2 complementing the flowering delay observed in the *ing2-2* mutant (Supplementary Fig. S3), previously employed for our AP-MS analysis (Table 1). ChIP-qPCR analysis showed an enrichment in the myc-ING2 immunoprecipitated fraction of selected amplicons in *FT* and *SOC1* loci coinciding with reported histone H3K4me3-enriched regions (Zhu et al. 2024). These observations indicate a direct binding of ING2 to these master regulator genes of flowering (Fig. 6B). In summary, our results support that ING2 directly regulates the transcript levels of *FT* and *SOC1* floral regulators by controlling their histone H4 acetylation levels.

## Discussion

PHD finger proteins epigenetically orchestrate different cellular processes in eukaryotes (Sanchez and Zhou 2011). To dissect the functional role exerted by plant PHD-containing ING proteins we have focused on the Arabidopsis ING2 protein, identifying co-immunoprecipitated proteins, analyzing the relationship with Arabidopsis ING1, and characterizing the physiological and molecular function of ING2. Our results establish ING2 as a bona-fide component of NuA4-C in plants, regulating a variety of developmental processes, including flowering time through the modulation of histone H4 acetylation at loci encoding key floral integrators such as *FT* and *SOC1*.

Our AP-MS analysis detected peptides corresponding to 8 out of 12 subunits that putatively constitute the plant NuA4-C (Espinosa-Cores et al. 2020). Together with data from additional AP-MS studies using different NuA4-C subunits as baits (Bieluszewski et al. 2015; Tan et al. 2018; Bieluszewski et al. 2022; Zhou et al. 2022; Zheng et al. 2023), as well as our previous protein–protein interaction results (Barrero-Gil et al. 2022), these observations provide solid evidence for ING2 as a core member of the plant NuA4-C (Table 1; Supplementary Fig. S1A). Unexpectedly, this experiment did not identify peptides corresponding to proteins such as Histone Deacetylase Complex 1 (HDC1), Sin3A-Associated Protein 18 (SAP18) or SHORT LIFE (SHL) that showed capacity to bind ING2 in tobacco transient co-expression experiments (Perrella et al. 2016). Similarly, the *Medicago* ING2 protein does not appear to interact with them either in yeast-two-hybrid assays (Jaudal et al. 2022). It is possible that the experimental conditions used in our study favor ING2 interaction with the NuA4-C. Alternatively, perhaps the recombinant *ING2* gene construct we used, though able to complement the phenotypic defects in *ing2* mutant plants, affects the capacity of tagged ING2 to interact effectively with HDACs. Intriguingly, we also identified in our AP-MS analysis peptides corresponding to proteins involved in pre-mRNA splicing (Chanarat and Strasser 2013) as well as proteins from the Mediator complex (Buendia-Monreal and Gillmor 2016) (Supplementary Fig. S1B), suggesting the ING2 function might be also linked to transcription initiation or mRNA splicing processes, an issue that will require further investigation.

Similar proteomic approaches using ING1 as bait have failed to identify NuA4-C subunits (Wu et al. 2023). Conversely, AP-MS studies using different NuA4-C subunits as baits have repeatedly failed to identify peptides corresponding to the ING1 protein. These observations suggest that ING1 and ING2 proteins might enable H3K4me3 reading in different chromatin-modifying complexes. In this context, while the Arabidopsis *ing2* mutant is characteristically late flowering (Fig. 1, A and B), we (Fig. 2, A and B) and others (Wu et al. 2023) could not identify a similar phenotype for Arabidopsis *ing1* mutant. We performed a genetic interaction analysis to address the relationship between both genes and found that *ING2* functions are largely independent of ING1 activity in the control of flowering time and other developmental processes. Moreover, the transcriptome regulated by ING2 is closer to the NuA4-dependent transcriptome while the ING1-dependent transcriptome is more similar to PAGA-C-regulated gene expression profile (Fig. 3D and Fig. 3E). Interestingly, a phylogenetic analysis of plant genes encoding ING proteins reveals that ING1 and ING2 plant clades appeared early in the green lineage (Jaudal et al. 2022). All these observations support the notion that despite their relative sequence similarity and domain composition, ING1 and ING2 proteins likely belong to separate chromatin-modifying complexes in Arabidopsis. As shown in Fig. 3C, we observe a larger number of misregulated genes in the *ing1 ing2* double mutant than in the single *ing1* or *ing2* mutants. This enhanced transcriptional misregulation detected in the double mutant plants might be due to the magnifying effect that simultaneous loss of NuA4 and SAGA-like HAT complexes could have on plant gene expression. In fact, several studies in yeast have already highlighted the interplay between NuA4 and SAGA complexes in gene transcription through chromatin acetylation (Durant and Pugh 2006; Ginsburg et al. 2009; Bruzzone et al. 2018). Further studies will be needed in plants to fully understand possible relationships between histone H3 and H4 acetylation in the regulation of gene expression. Intriguingly, a recent report reveals stronger developmental defects in *Medicago* plants lacking both ING1 and ING2 function compared to single *Mting2* mutant plants (Mayo-Smith et al. 2025). We have not observed such an enhancement of *ing2* developmental defects by the *ing1* mutation in Arabidopsis (Fig. 2), suggesting that the functional relationship between plant ING proteins might be adapted to particular requirements in different plant species.

Our physiological analyses of ING2 function revealed that this protein is involved in various developmental processes including flowering time, chlorophyll accumulation, and flower and fruit development (Fig. 1 and 2 and Supplementary Fig. S4). These phenotypic alterations are reminiscent of a loss-of-function *ING2 medicago* mutant characterized (Jaudal et al. 2022), indicating that ING2 physiological roles may be conserved in plants. Several mutant plants lacking the function of diverse NuA4-C subunits exhibit either an acceleration (Xiao et al. 2013; Bieluszewski et al. 2015; Gomez-Zambrano et al. 2018; Crevillen et al. 2019) or a delay of flowering time (Bu et al. 2014; Xu et al. 2014; Barrero-Gil et al. 2021). In our current study, we observed that the ING2 protein is a promoter of flowering in photoperiod-inductive conditions (Fig. 1A) and becomes essential for flowering under non-inductive conditions (Fig. 1B) (Fig. 2). We revealed that the ING2 protein activates the expression of *FT* and *SOC1* promoting histone H4ac levels at these chromatin loci (Fig. 6B). Moreover, our profile of histone H4ac in WT and *ing2* mutant plants demonstrates that such alterations in histone H4ac are not restricted to these genes but rather can be found genome-wide (Fig. 5A), including important flowering time regulators such as *GI* and *CO.* These genes act upstream of the floral integrators *FT* and *SOC1* and may also contribute to the observed mis-expression of these genes. Hence, we conclude that ING2 chromatin reader modulates histone H4ac levels and that alteration in its activity correlate with changes in gene expression (Fig. 5B). Intriguingly, we observe more genes differentially upregulated than downregulated in the *ing2* mutant (Fig. 3C), a trend also reported in other NuA4-C mutants such as *mrg1 mrg2* and *epl1ab* (see (Barrero-Gil et al. 2021, 2022), respectively). Further research is needed to precisely determine how defects on NuA4-C activity affect gene expression.

ING proteins have been shown to specifically recognize and bind H3K4me3 in vitro through their PHD domain (Lee et al. 2009). To gather further insights into the activity of ING2 as a H3K4me3 reader, we analyzed whether ING2-mediated flowering time was dependent on the function of H3K4me3 writers ATX1 and ATXR7 (Pien et al. 2008; Tamada et al. 2009). The results reveal an epistatic relationship between these genes regarding the regulation of flowering time (Fig. 4, A and B), which is consistent with a functional link between H3K4me3 deposition and ING2 activity that mediate NuA4-C acetylation on target genes bearing particular chromatin conformations or histone marks. Previous observations with the ING4 homolog, a subunit of the versatile HAT HBO1, support a crosstalk between histone H3K4 trimethylation and H3ac to attenuate cellular neoplastic transformation in metazoans (Hung et al. 2009). Further experiments are required, however, to elucidate the dependence of ING2 function on the deposition of H3K4me2/me3 and identify the molecular determinants involved as well as the relationship of these marks with histone H4ac. Finally, we have also explored a functional relationship between ING2 and SWR1-C activity (Figs. 4 and 5). Our genetic analysis revealed the dependence of *ing2* late flowering phenotype on SWR1-C subunits (Fig. 4E). Intriguingly, though previous works revealed a correlation between the NuA4-C activity mediated by EPL1 and H2A.Z global levels (Bieluszewski et al. 2022), we did not find any significant alterations in genome-wide deposition of the histone variant H2A.Z in the *ing2* mutant but rather, a limited effect of ING2 regulating the levels of H2A.Z that, intriguingly, appear to be weakly associated with histone H4ac (Fig. 5, Supplementary Fig. S9). Perhaps, ING2 is required for NuA4-C-regulated H2A.Z occupancy in a restricted subset of genes and the affectation of global histone H2A.Z requires higher levels of inhibition of NuA4-C activity. Alternatively, the observed effects of the loss of ING2 function on H2A.Z levels in the chromatin could be indirect, involving additional chromatin regulators. In addition, the effect of ING2 function on H2A.Z PTMs remains to be explored. In conclusion, the relationship between histone H4ac and H2A.Z occupancy in the *ing2* mutant is more complex than anticipated and further research is required to elucidate the molecular determinants underpinning the observed genetic interaction between ING2 and SWC6. An integration of our results with available genome-wide H3K4me3 data (Xu et al. 2025) indicates that ING2 preferentially controls histone H4 acetylation in highly H3K4me3-enriched genes (Supplementary Fig. S7A), which is consistent with its putative role as a H3K4me3 reader.

Our data support a working model (Fig. 6C) in which the ING2 subunit likely enables NuA4-C H3K4me3 reading and participates in the modulation of histone H4ac levels on underlying genes. This activity is required for several developmental processes, mainly the initiation of flowering, as well as flower and fruit development or chlorophyll accumulation. Importantly, ING2 function is largely independent of ING1 protein and does not seem to contribute significantly to the deposition of the histone variant H2A.Z. Further study is needed to dissect the molecular basis of the genetic interaction between *ING2* and genes encoding key subunits for the SWR1-C identified in our study.

## Materials and methods

### Plant materials, phenotypic analysis and growth conditions

All Arabidopsis lines used in this study were in Columbia-0 background. The mutant alleles *ing2-1* (GABI_166D07) and *ing2-2* (GABI_909H04) were obtained from the Nottingham Arabidopsis Stock Centre (NASC, UK). Other mutants used in this study have been previously described elsewhere: *ft-10* (Yoo et al. 2005), *soc1-2* (Lee et al. 2000), *atx1-2* (Pien et al. 2008), *atxr7*-2 (Tamada et al. 2009), *ing1-1* (Wu et al. 2023) and *swc6-1* (Lazaro et al. 2008). To generate *ing2* complemented lines we amplified *ING2* open reading frame with the primers we indicated in Supplementary Table S5 and cloned it in a pGWB18 expression vector (Nakagawa et al. 2007) that was transformed into *ing2-2* mutant plants by the floral dip method (Clough and Bent 1998). Unless otherwise indicated, plants were grown at 22 °C under LD photoperiods (16 h of cool-white fluorescent light) with photon flux of 100 *μ*mol m^−2^ s^−1^, in pots containing a mixture of organic substrate and vermiculite (3:1, v/v), or in Petri dishes containing 1/2x Murashige and Skoog (MS) medium supplemented with 1% sucrose where indicated, and solidified with 0.8% (w/v) plant agar.

Flowering time was determined by counting the total leaf number at the time of first flower opening (Lazaro et al. 2008) under both LD and SD photoperiods. Chlorophyll extraction was performed by overnight incubation of grounded tissue in N,N, dimethylformamide. Absorbance measurements at a wavelength of 647 and 664 nm of cleared extracts were taken on a C7100 spectrophotometer (PEAK Instruments) and chlorophyll concentration was calculated as described (Porra 2002).

### AP-MS analysis

Arabidopsis *ing2* mutant plants complemented with the 4xmyc-ING2 construct were generated (Supplementary Fig. S3). For that, the *ING2* coding sequence was mobilized to the destination vector pGWB18 (Nakagawa et al. 2007), which bears the constitutive CaMV 35S promoter and a 4xMyc epitope in N-terminal, by a recombination reaction with LR clonase I (Invitrogen). The resulting clone was checked with restriction enzymes and transformed into competent *Agrobacterium tumefaciens* AGL0 cells to produce transgenic 4xmyc-ING2 *ing2* plants that fully rescued the WT flowering time and were used for AP-MS analysis. Affinity purification was performed as described (Gomez-Zambrano et al. 2018) with slight modifications. Protein extraction was performed in extraction buffer (20 mm TrisHCl pH 8, 150 mm NaCl, 2.5 mm EDTA, 33 mm β-mercaptoethanol, 10% Glycerol and 0.5% Triton X-100 supplemented with cOmplete Protease Inhibitor Cocktail (Roche, Switzerland)) from 5 g of 12 day-old seedlings grown on Petri dishes. Extracts were incubated with μMACS anti-myc microbeads (Miltenyi Biotec, 130-091-123) for one hour at 4 °C before recovering immune-complexes. For analysis of ING2-bound proteins from pull-down experiments, proteins were digested as described (Gomez-Zambrano et al. 2018), with minor modifications. After digestion, tryptic peptides were analyzed by LC-MS/MS in an Orbitrap Fusion mass spectrometer (Thermo Fisher Scientific). An enhanced FT-resolution spectrum (resolution = 70,000) followed by the HCD MS/MS spectra from the n-th most intense parent ions were analyzed along the chromatographic run. Dynamic exclusion was set at 40 s.

For peptide identification, spectra were analyzed with Proteome Discoverer v2.1.0.81 (Thermo Fisher Scientific) using SEQUEST-HT engine (Thermo Fisher Scientific). Database search was performed against the Uniprot database containing all sequences from *Arabidopsis thaliana* and crap contaminants (December 5th, 2016; 77084 sequences), using the following parameters: trypsin digestion with 2 maximum missed cleavage sites, precursor and fragment mass tolerances of 800 ppm and 1.2 Da respectively, carbamidomethyl cysteine as fixed modification and methionine oxidation as variable modifications. Scaffold program (version 5.3.3, Proteome Software Inc., Portland, OR) was used to validate MS/MS based peptide and protein identifications, with an additional filter for precursor mass tolerance of 15 ppm (Bonzon-Kulichenko et al. 2015). Protein probabilities were assigned by the Protein Prophet algorithm (Nesvizhskii et al. 2003). We performed two independent pull-down biological replicates and the corresponding control protein purification from WT, and the identified peptides are listed in Supplementary Table S1.

### Gene expression analysis

For qPCR analysis, RNA was extracted from plants grown on the indicated conditions with the E.Z.N.A Plant RNA kit (Omega Bio-Tek). RNA was retro-transcribed using the Maxima first strand cDNA synthesis kit (Thermo Fisher Scientific). qPCR was performed using LightCycler 480 SYBR Green I (Roche). Primers used for qPCR analysis are listed on Supplementary Table S5. The *PP2AA3* (At1g13320) gene was used as a reference in all experiments.

For RNA sequencing experiments, 10 day-old seedlings were grown on petri dishes and plant tissue was harvested at ZT16. RNA was extracted from two independent experiments to prepare sequencing libraries for each genotype. RNA library preparation and sequencing was performed by Beijing Genome Institute (BGI) on a HiSeq2500 platform generating 4Gb clean 150PE reads per sample. Clean reads were mapped to Arabidopsis TAIR10 reference genome using HISAT2 v2.0.5 (Kim et al. 2015). Differential gene expression analysis was performed using the DESeq2 module from SeqMonk v1.35 software (Babraham Institute). DEGs were defined by FDR ≤ 0.05 and log2 fold change ≥1 or ≤ −1. GO enrichment analysis was performed on PANTHER (http://pantherdb.org/) using a Fisher's exact test corrected by a false discovery rate FDR < 0.05 as cutoff for a significantly enriched GO term.

### Chromatin immunoprecipitation

For ChIP experiments we collected aerial tissue from soil-grown plants grown for 14 days at 22 °C in LD conditions. Tissue was cross-linked with 1% formaldehyde for 10 min. Dual crosslinking was implemented for tissue from complemented myc-ING2 transgenic lines by incubating plant material for 30 min in the presence of 2 mm disuccinimidyl glutarate (DSG) followed by a 10 min incubation with 1% formaldehyde. Chromatin was isolated essentially as described (Komar et al. 2016). Histones were immunoprecipitated using the following antibodies: α-H4K5,8,12,16 ac (Millipore, 06-598), α-H4 (Millipore, 05-858), α-H2A.Z (Agrisera, AS10718) while 4xmyc-ING2 was immunoprecipitated with, α-myc (Millipore, 05-724). For ChIP-qPCR experiments cross-links were reversed by a 10 min incubation at 95 °C in the presence of 10% Chelex-100 resin (Biorad) followed by Proteinase-K treatment. For the ChIP-seq experiment we reversed cross-links by incubating chromatin overnight in 0.2 m NaCl at 65 °C followed by Proteinase-K treatment. Two libraries per genotype and antibody were prepared using QIAseq Ultralow input library kit (QIAgen) and sequencing was performed by Novogene (https://www.novogene.com) in a Novaseq X Plus instrument generating 6 Gb of 150PE reads. Clean reads were mapped to Arabidopsis TAIR10 reference genome using bowtie2 v2.3.5.1. Metaplots were generated using deepTools v3.5.4. Enriched peaks were called using MACS2 v2.2.6 and differential peak analysis was performed using the bdgdiff module from this program.

### Accession numbers

The complete sequencing data from this publication has been submitted to the Sequence Read Archive (SRA) database (www.ncbi.nlm.nih.gov/sra/) and can be accessed under accession number PRJNA1235187.

Accession numbers for the genes investigated in this study can be found in Table 1 and Supplementary Table S6.

## Supplementary Material

kiaf511_Supplementary_Data

## Data Availability

The data underlying this article are available in the article and its online Supplementary material. Sequencing data from this article have been deposited in the Sequence Read Archive (SRA) database (www.ncbi.nlm.nih.gov/sra/) under the accession number PRJNA1235187.

## Supplementary data

The following materials are available in the online version of this article.

**Supplementary Figure S1.** Interaction of ING2 with NuA4-C subunits.

**Supplementary Figure S2.** Validation of *ING2* T-DNA insertion lines used in this study.

**Supplementary Figure S3.** A myc-tagged ING2 construct complements the late flowering displayed by the *ing2* mutant.

**Supplementary Figure S4.**  *ing1 ing2* double mutants show an enhancement of fruit developmental defects.

**Supplementary Figure S5.** Gene ontology enrichment analysis on differentially expressed genes in *ing* mutants.

**Supplementary Figure S6.** Differences in histone H4 occupancy do not explain the lower levels of histone H4ac found in the *ing2* mutant.

**Supplementary Figure S7.** ING2-dependent H4 acetylation is higher in H3K4me3-enriched genes.

**Supplementary Figure S8.** Loss of *ING2* function does not change histone variant H2A.Z levels at *FT* and *SOC1* genomic loci.

**Supplementary Figure S9.** Relationship between histone H2A.Z occupancy and ING2-mediated histone H4 acetylation.

**Supplementary Table S1.** Proteins co-purifying with ING2 in AP-MS experiments identified with Scaffold5 software.

**Supplementary Table S2.** List of differentially expressed genes (DEGs) in *ing1*, *ing2* and *ing1 ing2* mutants compared to wild type.

**Supplementary Table S3.** Genes with decreased H4ac levels in the *ing2* mutant.

**Supplementary Table S4.** Genes with altered levels of H2A.Z occupancy in the *ing2* mutant.

**Supplementary Table S5.** Primers used in this study.

**Supplementary Table S6.** Accession numbers for the genes investigated in this study.

## Dive Curated Terms

The following phenotypic, genotypic, and functional terms are of significance to the work described in this paper:

ING2 Gramene: AT1G54390
ING2 Araport: AT1G54390

ING1 Gramene: AT3G24010
ING1 Araport: AT3G24010

HAM1 Gramene: AT5G64610
HAM1 Araport: AT5G64610

PP2AA3 Gramene: AT1G13320
PP2AA3 Araport: AT1G13320

ARP4 Gramene: AT1G18450
ARP4 Araport: AT1G18450

EAF6 Gramene: AT4G14385
EAF6 Araport: AT4G14385

SAP18 Gramene: AT2G45640
SAP18 Araport: AT2G45640

FLC Gramene: AT5G10140
FLC Araport: AT5G10140

EPL1B Gramene: AT1G79020
EPL1B Araport: AT1G79020

ATX1 Gramene: AT2G31650
ATX1 Araport: AT2G31650

ATXR7 Gramene: AT5G42400
ATXR7 Araport: AT5G42400

FT Gramene: AT1G65480
FT Araport: AT1G65480

SOC1 Gramene: AT2G45660
SOC1 Araport: AT2G45660

SWC6 Gramene: AT5G37055
SWC6 Araport: AT5G37055
