# Supplementary material for: Arabidopsis INHIBITOR OF GROWTH 2 promotes flowering by regulating NuA4-dependent H4 acetylation levels at FT and SOC1
Source: Plant Physiol. 2025 Oct 16;199(3):kiaf511. doi: 10.1093/plphys/kiaf511 (PMC12579105; doi:10.1093/plphys/kiaf511)
Supplement: kiaf511_Supplementary_Data [file kiaf511_supplementary_data.zip › Supplementary_Data.pdf]

SUPPLEMENTARY DATA

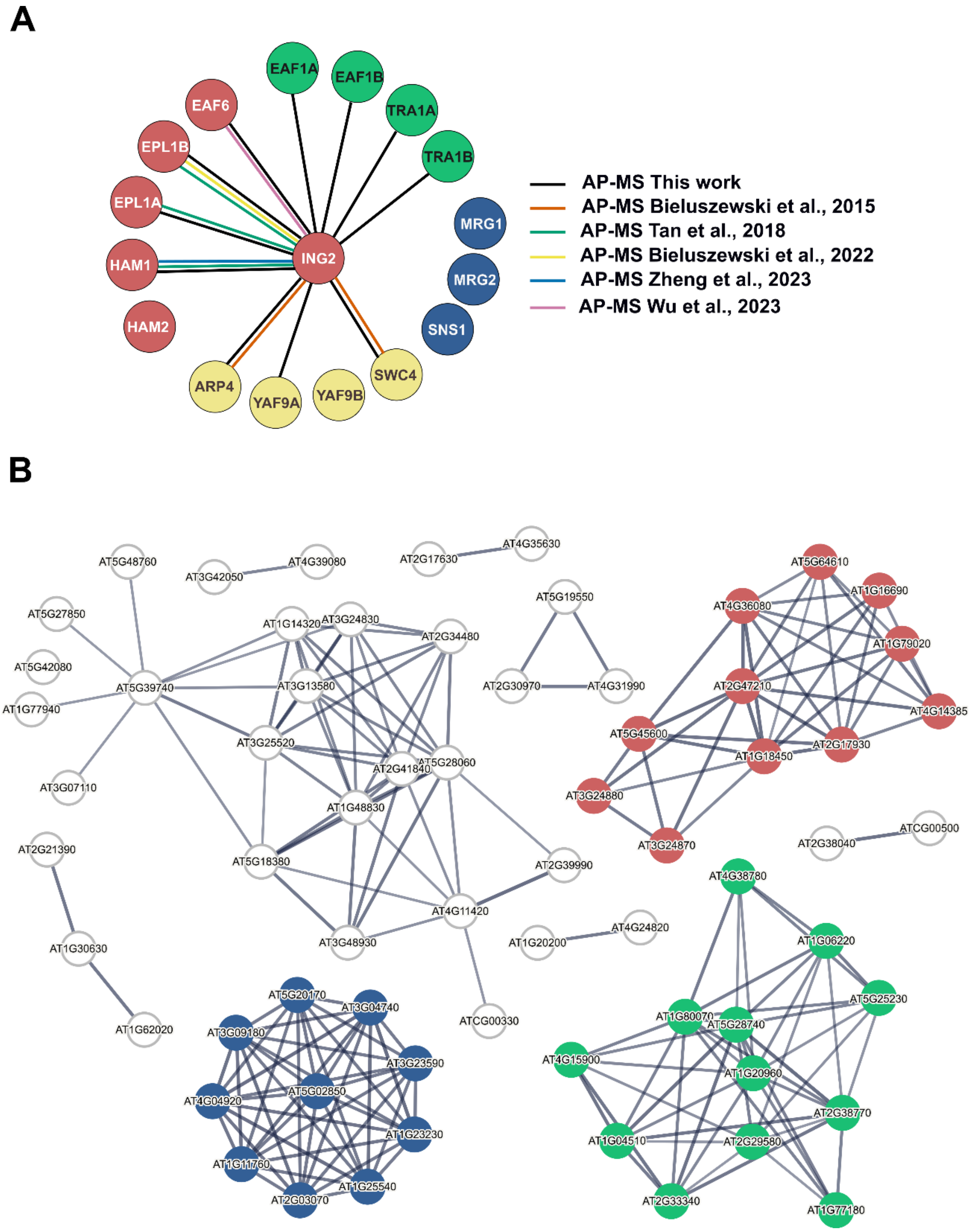

Supplementary Figure S1: Interaction of ING2 with NuA4-C subunits. (A) Schematic diagram showing current AP-MS evidence supporting ING2 interaction with NuA4-C subunits. Circle colors indicate NuA4-C modules: piccolo (red), core (green), TINTIN module (blue), and YEATS (ochre). Line colors indicate AP-MS experiments and the corresponding references. (B) Functional

protein networks identified among ING2-copurifying proteins. PRP19, Mediator and NuA4 complexes have been highlighted in green, blue and red, respectively.

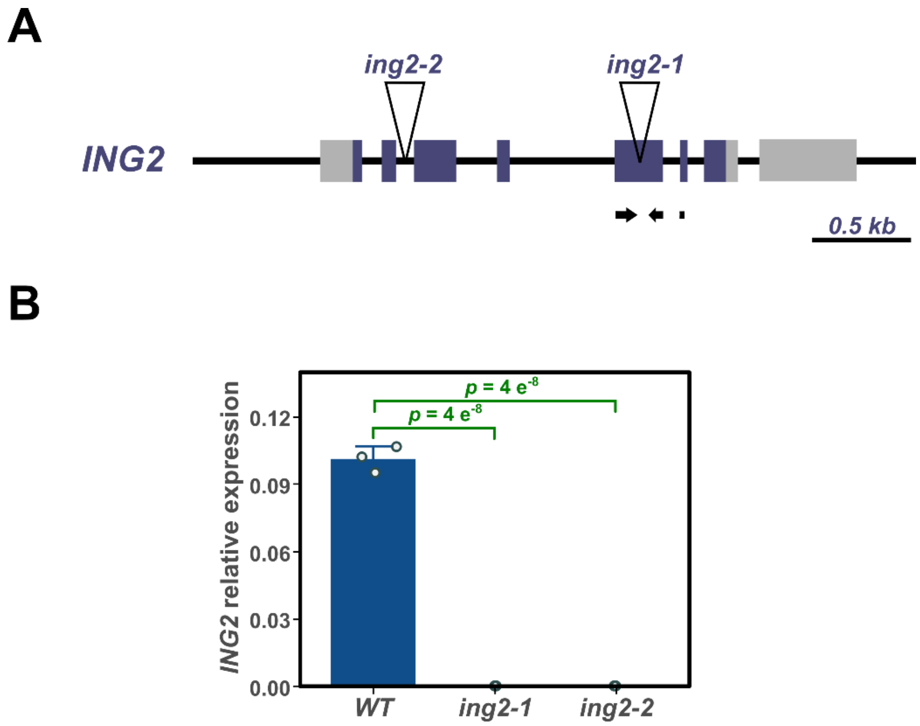

Supplementary Figure S2: Validation of *ING2* T-DNA insertion lines used in this study (A) Schematic representation of the *ING2* gene showing the location of T-DNA insertions in *ing2-1* and *ing2-2* mutant lines. Primers used to check *ING2* expression in T-DNA lines are indicated by arrows. (B) qPCR gene expression analysis in plants from the indicated genotypes. Bars and error bars indicate average and standard deviation from three biological replicates. Statistical significance is indicated providing *p*-value in a Dunnett's test.

A

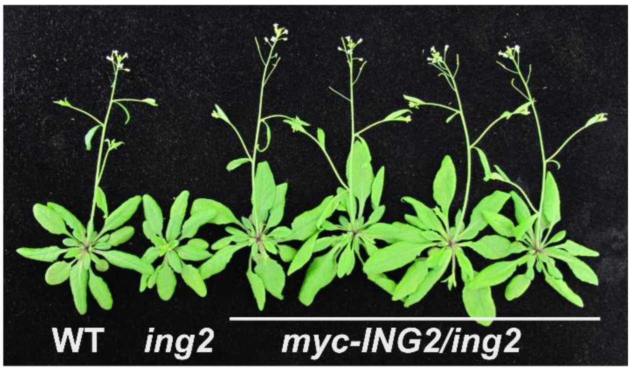

B

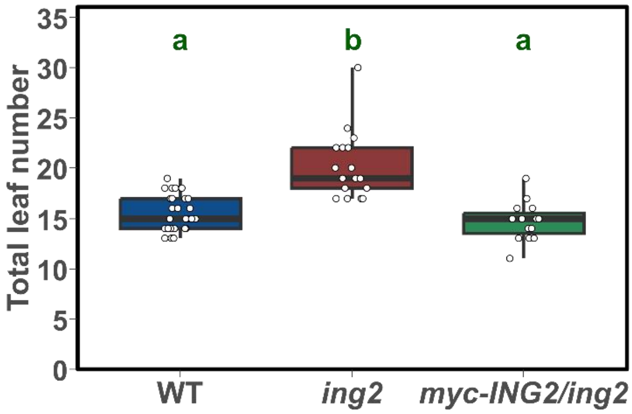

Supplementary Figure S3: A myc-tagged ING2 construct complements the late flowering displayed by the *ing2* mutant. (A) Representative plants of the indicated genotypes grown under LD conditions. (B) Determination of flowering time in the indicated genotypes. Box plots indicate interquartile range while a line indicates the median and whiskers represent minimum and maximum values. Points show individual observations. Statistical significance in a one-way ANOVA at $p < 0.05$ followed by Tukey's HSD test is indicated by different letters.

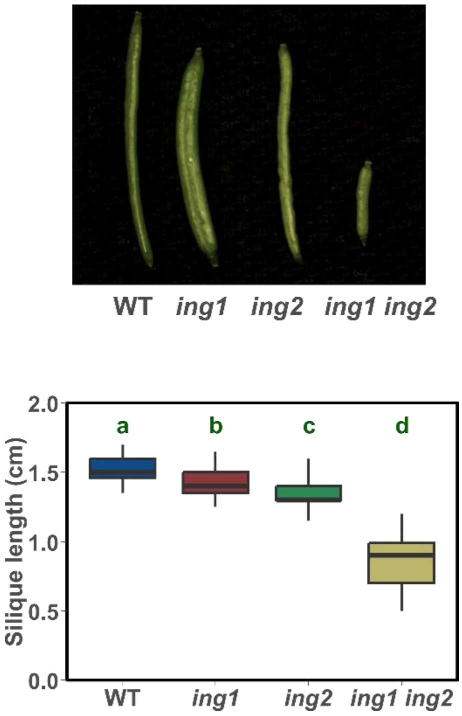

Supplementary Figure S4: *ing1 ing2* double mutants show an enhancement of fruit developmental defects. Fruit length in LD grown plants of the indicated genotypes. Box plots indicate interquartile range while a line indicates the median and whiskers represent minimum and maximum values. Statistical significance in a one-way ANOVA at $p < 0.05$ followed by Tukey's HSD test is indicated by different letters.

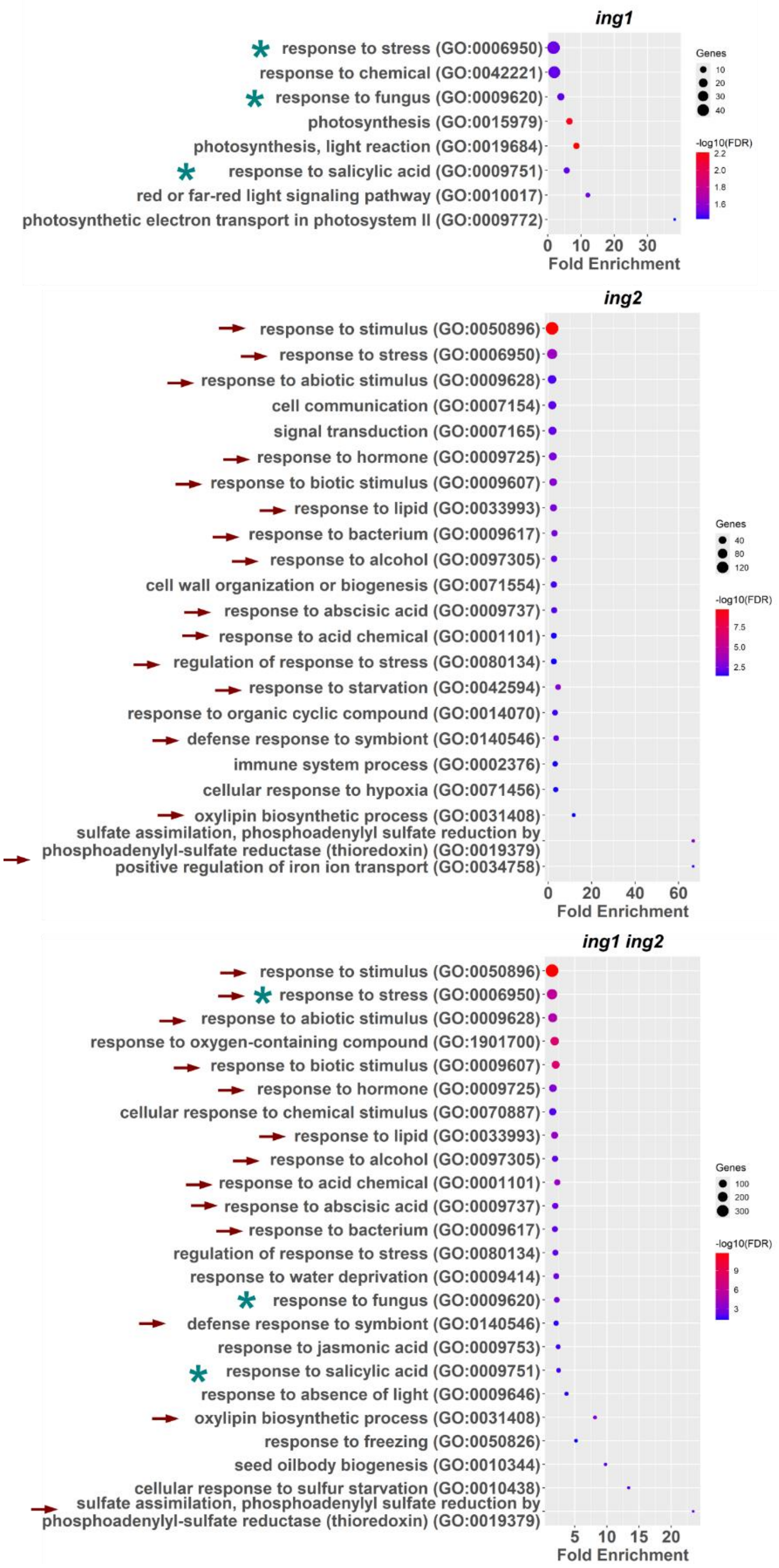

Supplementary Figure S5: Gene ontology enrichment analysis on differentially expressed genes in *ing* mutants. Selected enriched GO terms (biological processes category) among identified DEGs in *ing* mutants. Shared GO terms between *ing1 ing2* and *ing1* (blue asterisks) or *ing2* mutants (red arrows) are indicated.

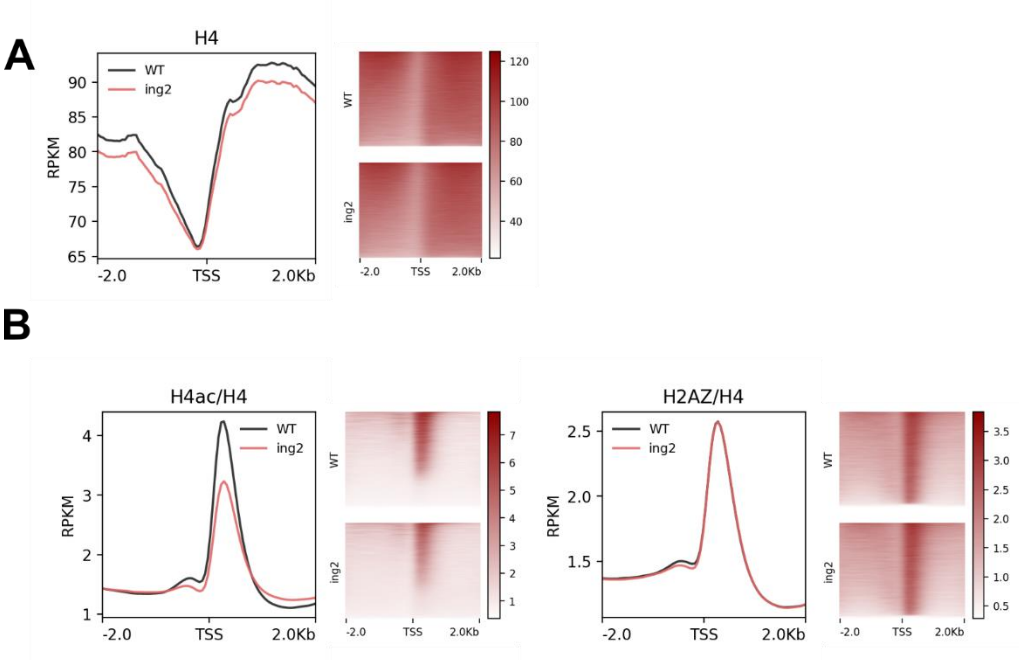

Supplementary Figure S6: Differences in histone H4 occupancy do not explain the lower levels of histone H4ac found in the *ing2* mutant. (A) TSS-centered profile and read density map displaying histone H4 occupancy levels in WT and *ing2* mutant. (B) Metaplots and read density maps showing histone H4-normalized levels of H4ac and H2A.Z in WT and *ing2* mutant.

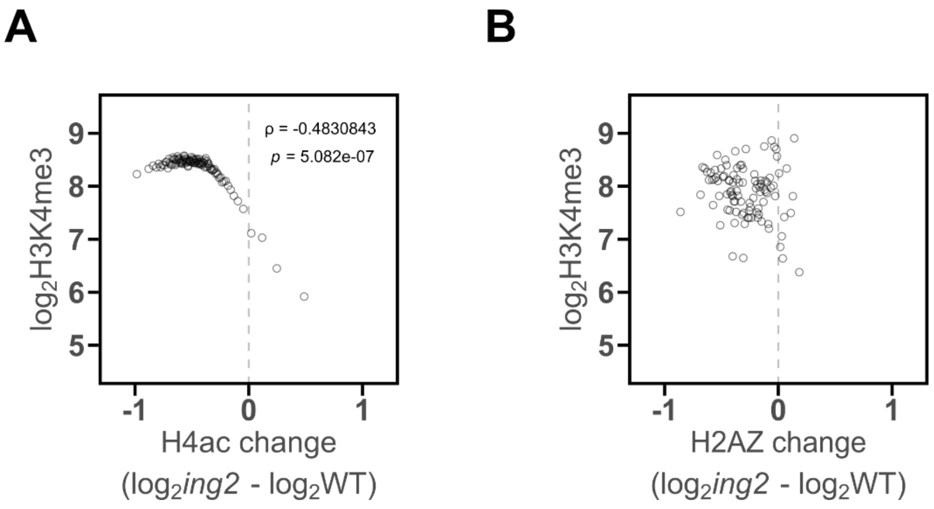

Supplementary Figure S7: ING2-dependent H4 acetylation is higher in H3K4me3-enriched genes. (A-B) Spearman rank correlation analysis between differential histone H4ac (A) or H2A.Z (B) in the *ing2* mutant and histone H3K4me3 signal level in wild type plants. The signal for histone H4ac (A) and H2A.Z (B) over a region spanning 500 base pairs downstream of the TSS was averaged for each gene identified by differential peak enrichment analysis. For each gene we also obtained a similar averaged histone H3K4me3 signal from a previous report (Xu et al., 2025). Spearman coefficient and *p*-value are indicated in the inset within scatter plot in (A).

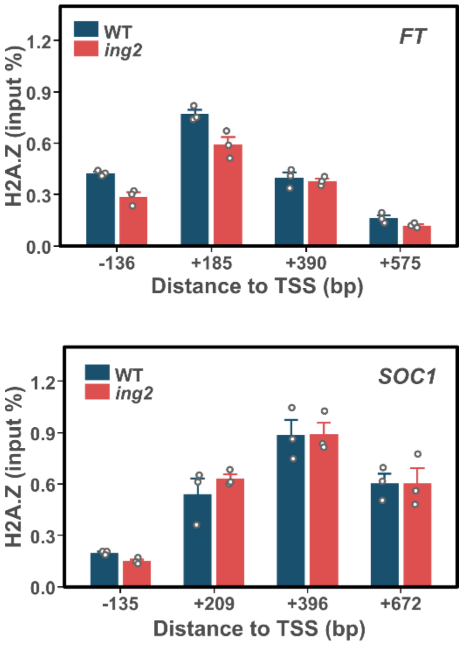

Supplementary Figure S8: Loss of *ING2* function does not change histone variant H2A.Z levels at *FT* and *SOC1* genomic loci. Histone H2A.Z levels at *FT* and *SOC1* genomic loci as determined by ChIP-qPCR experiments testing the same amplicons indicated in Figure 6. Bars indicate the average and error bars denote the standard deviation from three biological replicates.

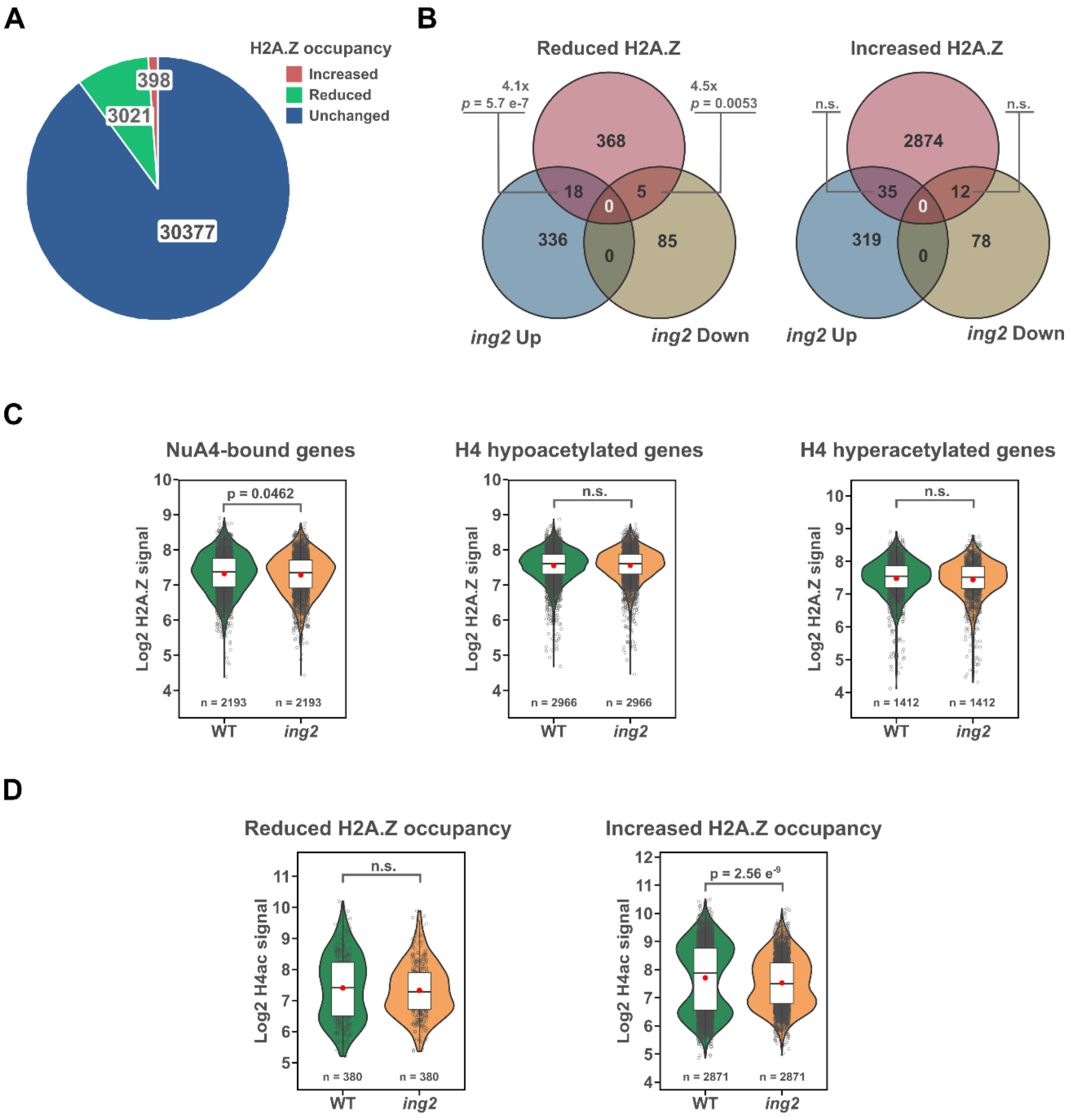

Supplementary Figure S9: Relationship between histone H2A.Z occupancy and ING2-mediated histone H4 acetylation. (A) Changes in histone H2A.Z occupancy in the *ing2* mutant. (B) Overlap of genes with altered histone H2A.Z occupancy and expression in the *ing2* mutant. Significant enrichment and the probability to find the observed overlap or higher in a hypergeometric test are indicated (n.s., not significant) (C) Histone H2A.Z signal over the first 500 bp downstream of TSS in loci related to ING2-regulated NuA4 activity. NuA4-bound genes are defined as loci bound by EPL1 and HAM1 as shown by Wu et al. 2023. (D) Histone H4ac signal over the first 500 bp downstream of TSS in loci identified by differential histone H2A.Z occupancy peak analysis. Boxes in boxplots show interquartile range, a line shows the median and a red dot indicates the average.

Brackets in C-D designate Mann-Whitney U tests to compare distributions (n.s., not significant).
